# Work related musculoskeletal injuries sustained by Australian osteopaths: qualitative analysis of effects on practitioner health, clinical practice, and patient care

**DOI:** 10.1186/s12998-017-0158-7

**Published:** 2017-10-03

**Authors:** Gopi Anne McLeod, Katerina Annels, Jessica Cohen, Samuel Edwards, Daniel Hodgins, Brett Vaughan

**Affiliations:** 1 School of Health & Human Sciences, Southern Cross University, Lismore, Australia; 2 School of Nursing & Midwifery, Central Queensland University, Noosa, Australia; 3 School of Health & Biomedicine, Victoria University, Melbourne, Australia

**Keywords:** Work-related musculoskeletal injuries, Osteopath, Osteopathic medicine, Osteopathy education

## Abstract

**Background:**

There is limited literature that explores the experiences of osteopaths injured while engaging in clinical practice. Evidence from other similar health professions has described the numerous effects of work-related musculoskeletal injuries (WRMI). Work-related musculoskeletal injury refers to trauma to joints, ligaments, muscles and tendons resulting from injury sustained while undertaking work duties. This research aimed to gain a contextualised understanding of the experiences of osteopaths who have sustained a work-related musculoskeletal injury while performing clinical practice.

**Method:**

This research used a descriptive qualitative design. Participants were recruited as part of a larger cross-sectional study. Thirteen Australian osteopaths who had sustained a work-related musculoskeletal injury consented to participate in semi-structured interviews during May and June 2016. Thematic analysis was used to elicit important themes from the interview transcripts that had been recorded and transcribed verbatim. The qualitative accounts provided by the participants were coded for the impacts of their injuries on work, home life and leisure activities.

**Results:**

The participants provided detailed, contextual information about their injuries, including the contributing factors and the experience of living with a WRMI. The findings indicate that injured osteopaths often continue working because of financial commitments and their dedication to patient care. The participants offered insights into the challenges they faced due to the injury and the management strategies they used to deal with the impact on their work and personal life. The injuries were mostly unreported, the burden being carried by the participants and their families.

**Conclusion:**

This is the first research that explores the experiences of osteopaths who have sustained a WRMI. We anticipate that this research will encourage a broad and constructive discussion within the profession of the issues associated with WRMIs, including risk minimisation and injury prevention. Further research is warranted to understand the relationship between osteopaths training in ergonomics and injury prevention. This would lead to the development of guidelines and educational curricula addressing safe work for osteopaths.

## Background

Work-related musculoskeletal injuries (WRMIs) are prevalent among health professionals who perform manual therapy, with less-experienced workers at greater risk of injury [1–5]. Work-related musculoskeletal injuries refer to trauma to joints, ligaments, muscles and tendons resulting from injury sustained whilst undertaking work duties. Chiropractors, physiotherapists (PTs) and occupational therapists (OTs) who have sustained a WRMI have reported negative impacts for patients and the practitioner themselves. Practitioners report being physically unable, or have a limited capacity, to perform certain techniques or work their usual hours with patients consequently receiving suboptimal care [2, 6–8]. The impact on a practitioner has also been reported to extend to impacting their home life [7].

Multiple risk factors that may lead to an increased risk of a WRMI have been identified. In one qualitative study involving 18 Australian PTs who had sustained a WRMI, participants indicated that due to a perceived moral obligation to be knowledgeable and caring, they felt pressured to perform techniques they knew held a risk of injury to themselves [9]. Such pressure has been interpreted by some authors as a risk factor for injury [10]. Other risk factors identified by researchers include treating a large number of patients in a single day, repetitive technique performance, responding to unexpected patient movements, working in cramped spaces, work postures and working whilst injured [11–13]. Graham and Gray [10] conducted focus group interviews with PTs to explore practitioners’ attitudes towards their personal risk of developing a WRMI. These authors found that practitioners continued to use a certain patient intervention despite knowing it was putting their health at risk. A systematic review by Anderson and Oakman [14] identified that female gender, work environment (particularly acute patient or outpatient settings), age, and potentially psychosocial factors such as job stress may also be risk factors for WRMIs.

A recent survey of Australian osteopaths revealed that WRMIs are widespread in the profession, with almost two-thirds of the participants reporting one or more injuries and more than half of the reported injuries occurring within the first five years of practice (McLeod G, Murphy M, Henare T, Dlabik B: Prevalence of work-related injuries in osteopaths: A preliminary investigation, submitted). This is consistent with research conducted by Glover et al. [2] with PTs in the United Kingdom and with the research of Holm and Rose [5] with chiropractors in America. The twelve month prevalence of WRMIs for the allied health workforce appears to be between 21% and 96% [2, 15–17].

Most research studies examining WRMIs in manual therapists have used questionnaires to estimate prevalence and identify body part injured, but there is limited qualitative research that explores practitioners’ experiences of their injuries. Apart from the findings of limited survey research, little is known about the experiences and perceptions of osteopaths who have been injured at work. This research aimed to gain a deeper understanding of the perceptions and experiences of osteopaths who have become injured at work, including the effects on their work and personal life; how they managed their WRMI(s); and the injury prevention suggestions they offer the profession.

## Methods

### Research design

This research employed a descriptive qualitative design. Semi-structured interviews were used to explore osteopaths’ experiences of becoming injured at work, strategies used to manage their WRMI(s) and to explore their suggestions for injury prevention in osteopaths. The current paper forms part of a larger cross-sectional study describing the prevalence and type of WRMIs in Australian osteopaths. This research was approved by the Southern Cross University Human Research Ethics Committee, approval number ECN-15-226.

### Participants

Participants were volunteers recruited from a national survey of Australian osteopaths, part of the larger study mentioned above. Of the 160 osteopaths who responded to the national survey, 19 indicated a willingness to participate in an interview. Contact was attempted with all 19 osteopaths. Of the 15 who were contactable, two were no longer available for interview. All of the remaining 13 consented to an interview.

### Ethics, consent and permissions

Preceding each interview, participants received information that outlined the aims of the research and how information gathered during the interview would be used. Participants were informed that all information gathered would be anonymous and any data used in reporting the research would be de-identified to protect participant privacy. Written consent was obtained from each participant.

### Data collection

Semi-structured interviews, ranging from fifty to sixty-five minutes, were conducted by three members (K.A. J.C. & S.E.) of the research team. The interviews took place between December 2015 and June 2016. The research team developed interview questions to address issues considered important based on the results of previous WRMI research in manual therapists [7, 18, 19]. Table 1 shows the topic guide used to develop the interview question protocol. The interviews commenced with questions related to demographics and confirmation of information provided in a previous survey (reported separately). Interviewers had an interview question protocol (see Appendix); however, a semi-structured approach allowed interviewers to probe participant responses in order to gain a deeper understanding of participants’ experiences.

**Table 1:** Topic guide for interviews

Topic	Content and focus of topic
Demographics	Place and year of graduation, highest level of education, age, gender, work situation and hours of work per week, length of time in clinical practice (since graduation and before injury)
Mechanism of injury	Identify body part(s) injured and explore exposure factors related to how and why the injury occurred
Effects of injury	Uncover practitioner experiences of the effects on work practices, and home and leisure activities (including financial, psychological and social)
Coping with a WRMI including treatment	Managing effects of injury and exploring methods of rehabilitation
Suggestions	Participants’ suggestions for the profession of osteopathy, including prevention and risk minimisation of WRMIs

### Data analysis

Qualitative analysis of the data followed the thematic analysis framework described by Braun and Clarke [20]. Qualitative analysis is useful for understanding social phenomena by placing emphasis to the meanings, views and experiences of the participants [21]. Thematic analysis is a qualitative data analysis technique in which the researcher identifies patterns within the data from which a set of concepts are developed to explain the experiences of participants. The interviews were electronically recorded and transcribed verbatim. Each transcription was analysed thematically by three researchers (with all researchers analysing at least three transcripts) to ensure accuracy and completeness of interpretation. This process [20], allowed for the identification and interpretation of the participants’ meanings presented in the data. The three researchers independently analysed each of the transcripts to identify themes. Inconsistencies in the coding of the data were resolved through consensus.

## Results

Qualitative thematic analysis revealed that the osteopaths in this study were able to articulate clearly the various facets of their experience of having sustained a WRMI. The findings further suggest that the experience of a WRMI was, for these osteopaths, a challenge that prompted resilience and adaptation. Participants offered advice for injury prevention grounded in their experience both as a practising osteopath and from their own personal experience. We have first presented the demographic profile of the participants followed by the key themes that explore the participants’ experiences of WRMI. Quotes from the interviews are included to clarify and support our interpretation.

### Demographics

Of the 13 osteopaths interviewed, four were female and nine were male. Their ages ranged from 26 to 58 years. The number of years in osteopathic practice ranged from two to 38 years, with an average of 10.5 years. There was no consistency about the time in practice before sustaining an injury: three participants were injured during their first two years of practice, five had been in practice between five and 10 years before sustaining their injury, four had been in practice more than 10 years, and one participant had been in practice more than 20 years before injury.

Two-thirds of the participants reported sustaining more than one injury, with three reporting three or more individual WRMIs. These participants were encouraged in the interview to discuss the injury they perceived as the most serious and/or as having the most significant effect on work and their life in general.

### Qualitative findings from thematic analysis

The qualitative thematic analysis produced four main themes: theme two has two subthemes that describe risk factors and risk modification used by participants. The four main themes are:

1. Perseverance
2. Inevitable change
3. Seeking help
4. Injury prevention in osteopathy education

#### Theme 1: Perseverance

This theme exemplifies the participants’ resilience and their motivation to recognise and manage the impact of a WRMI on the different aspects of their life. The following provides a picture of the participants’ experiences of initial concern and subsequent drive to maintain, to some extent at least, the status quo of clinical practice.

The majority of participants clearly expressed their determination to continue working whilst injured, regardless of the limitations posed by their WRMI. Although they voiced concern that they might not be able to continue working in the same manner as before the WRMI, they explained that they needed to make changes that permitted them to continue to provide patient care and ensure the viability of their business.

*I didn’t know what to do for a while—I just wondered how I was going to keep on going. I was quite [worried] and I realised that I had to do something different. I had to change the way I was doing things. (Participant No. 13).*

There was, however, a tendency for some participants to just ‘push through’. When asked how they managed their injury at work, a few said they continued working despite their injuries and pain. “I’m the type of person that just pushes through things … using painkillers” (Participant No. 6). Many participants conveyed a high level of commitment to their work and to their patients’ health and wellbeing, often putting the patients’ health before their own. Taking time off work due to injury was not a popular option. For those who did take time off, they did so only when suffering considerable pain or disability.

#### Theme 2: Inevitable change

Work as an osteopath is demanding both physically and mentally due to the physical nature of the work and the entrusted responsibilities of patient care. This theme explores the participants’ experiences of the various risk factors inherent in clinical practice, the changes injury had on their practice and life and the changes they implemented to modify risk and manage WRMI. Participants discussed numerous factors that they thought contributed to their injury as well as the risk modification strategies that they instigated. There are two subthemes within this theme, (1) ergonomic and biomechanical factors: risk and risk modification, related to practitioner and equipment, and (2) psychosocial factors: risk and risk modification.

### Ergonomics and biomechanical factors: Risk and risk modification

Faulty practitioner ergonomics and posture was mentioned by participants with many believing it played a significant role in their injuries. Poor technique selection and execution, cumulative effects of repeated loading of joints and soft tissues and overuse were also identified by participants as risk factors. Responses varied between those who were recent graduates and those who had studied many years ago when ergonomics in practice had not been emphasised by their educational institution. There was also differences between the experiences of male and female participants.

For example, several of the male osteopaths had treated patients considerably larger than themselves and suggested that larger patients often sought treatment from a male practitioner. They believed their injury had resulted from the poor ergonomic positioning and extreme stretching of their own body, required to accommodate the patient’s size. Participant No. 13 explained that providing treatment for a “bigger patient, resulted in tension restriction around the diaphragm area”.

Participants also mentioned the effects of poor ergonomics in previous employment, as a risk factor to injury. They suggested that the accumulation of poor technique usage over time had either led to or compounded their injury. One participant (Participant No. 3) highlighted poor technique as a massage therapist prior to becoming an osteopath as a major contributing factor that ‘certainly had an effect’ on their current injury.

The following quotes highlight the influence of a previous work situation on injury.

*I was a chartered accountant before, sitting down all day and studying all night. I would say neck stiffness, particularly around the cervicothoracic junction, definitely contributed to my injury. (Participant No. 7).*

*[The injury was] originally just an overuse one; I was doing massage as a uni student. It was more like a tendonitis, like a flexor tendonitis through my elbow and wrist and a bit of carpel tunnel. [Participant No. 12].*

Numerous strategies were highlighted with awareness to posture as a primary preventative measure for WRMIs. For example, Participant No. 6 suggested injury prevention for her is “all about posture and how I’m actually standing and leaning over people”, and participant No. 2 explained: “I use self-postural awareness… a full-length mirror, so I can correct my posture whilst working”. Others discussed pre-empting overuse injuries by decreasing the number of repetitive or posturally demanding techniques in clinical practice.

Some participants attributed their injuries to a lack of physical fitness and strength. This was, for some, a “vicious circle” where injury caused weakness and weakness caused injury. They related how this played out in other areas of their lives besides work, such as driving, engaging in sport and fitness training and even getting out of bed in the morning. Participant No. 12 lamented that they were unable to do strength training since a WRMI and Participant No. 7 said, “I actually had to stop that [judo and jujitsu] a few years ago because I just couldn’t do the gripping anymore”.

Two female participants felt they had returned to work too soon after childbirth when their fitness and strength were reduced. Participant No. 4 explained that ‘not being as physically active and fatigue from a lack of sleep’ had led to her injury, and Participant 11 succinctly described that her return to clinical practice after childbirth was “too much too quickly, without any match fitness for treating”. This participant went on to explain that her early return from maternity leave meant she “didn't have as much core strength and was over recruiting my upper body”. Another female participant proposed that hormones related to menstruation increased the risk of injury.

Participants also discussed equipment that was not ergonomically appropriate for carrying out certain techniques. Using the correct treatment bench is important for osteopaths, and several study participants suggested that using a bench that was either too wide, too narrow or not height-adjustable was a major factor in the development of their injury. Participant No. 9, for example, recounted how as a beginner practitioner he did not have an adjustable-height bench. He believed that this was a major contributing factor to his injury because it necessitated “bending over benches in an awkward position”. Participant No. 6 suggested the width of her bench had contributed to her injury, stating, “it is a little too wide”. Many discussed how addressing treatment table height and width, determining a safe technique selection for each patient and requesting patients to move themselves during techniques had been critical to recovery and to prevention of further injury.

In addition to discussing the ergonomics of equipment and posture, there were specific techniques that some participants felt were *dangerous* due to the high compressive forces required for their execution. This included high-velocity low-amplitude (HVLA) techniques and myofascial work requiring sustained or repetitive force, such as rib-raising and cross-fibre massage. Although many practitioners considered soft tissue techniques an integral part of their diagnosis and treatment practices, they believed the repeated and sustained pressure of these techniques contributed to their injury. Repetitive strain and overuse were reported as common occurrences, not only in the wrists and hands but also in other parts of the body if a participant used the same technique frequently.

The HVLA techniques that participants identified as causative of injury were seated thoracic spine HVLA, lumbar spine HVLA and a supine thoracic spine HVLA known as a ‘cuddle’. Participant No. 6 described her experience of using HVLA to be “quite vigorous and compressive” and said she was “weaning” patients off this technique type.

Almost half of the participants identified the HVLA technique commonly called a “cuddle,” in which the practitioner uses an epigastric contact on the supine patient’s elbows [22], as the injurious technique. Although Participant No. 13 identified the ‘cuddle’ as their favourite spinal technique, he also said, “I usually regret it later”. Others also commented on this technique.

*I [sometimes] damaged my sternum when I used a cuddle technique. I would yelp, and patients would look at me and see I had gone white, and ask me if I was OK and if I needed to sit down. (Participant No. 9).*

*I have a mid-thoracic injury that I think is from doing cuddles. It’s not overuse but compression and flexion/ extension of the thoracic spine from doing that thrust. (Participant No. 8).*

Most participants said that techniques that caused injury needed to be either adapted or not used at all. This often involved modifying factors associated with ergonomic and biomechanical risks and reducing the frequency with which they were used.

*I cut back work, restricted treatments to one cuddle per patient and applied it with far less force. (Participant No. 8).*

*You are going to be susceptible to injury, and you just need to think it through biomechanically and, yeah, work out ways to minimise it. (Participant No. 13).*

Participant No. 1 also made the suggestion that “if you do acquire an injury, stop immediately and act early” and Participant No. 10 suggested, “it’s not worth the long-term costs of putting up with pain when you treat”. However, in contrast to these views, Participant No. 5 believed that injury was inevitable with certain techniques and one had to be ‘willing to pay the ultimate price, which is probably a chronic overuse injury’.

For the most part, the participants were certain of both the cause and the factors that had led to—and in some cases continued to aggravate—their injury. This finding is not surprising because osteopaths spend a great deal of their work time discussing and diagnosing the injuries of others. However, there were examples of participants who struggled to understand and manage their injury.

*It [the injury] would go away and then come back— Go away after I had a sleep and then come back again when I worked or did too much—and then go away again. Then it just stayed and got worse and then I also injured my left [wrist], because I was overusing my left hand. I was like okay! I had five months off work and used my income protection and that’s when I really started to investigate it. I went to the sports physician and he thought it was carpal tunnel. So, I had a cortisone injection and then I saw a hand specialist and he then thought it wasn’t carpal tunnel. He sent me to the neurologist. The neurologist did nerve conduction studies and MRI’ed my neck and didn’t find anything, (Participant No. 3).*

### Psychosocial factors: Risk and risk modification

Psychosocial factors can both contribute to and be the result of a WRMI [23]. Most of the osteopaths in this study were aware of the influence of psychological stress and the pressure of overwork on the risk of physical injury. Several participants discussed how they were more likely to be injured late on a workday when fatigued, especially when seeing a large number of patients. For example, Participant No. 1 acquired their injury when they were ‘seeing 60 to 80 patients a week’. It was suggested by Participant No. 6 that the reason her injury was worsening over time was due to “increased fatigue with the greater number of years in practice”.

For many, a WRMI meant reducing the time they spent in clinical practice. Some participants said they reduced their overall hours worked each week; others said they decreased the number of hours worked each day. This reduction in time spent in clinical practice resulted in a decrease in the number of patients seen. For some, a period off work was required.

*Since the diagnosis, I have reduced patients from 20 down to 14 patients a day and more breaks. I’m now doing three or four consultations and have a 30-min break. (Participant No. 7).*

Most participants spoke of the pervasive and detrimental influence of injury on their general health and wellbeing. Several participants discussed the frustration and distress they felt because the effects of WRMI forced them to abandon the sports and other activities that were central to their well-being. This included interacting and connecting with family and friends and involvement in activities such as yoga, martial arts, gym work, and sports such as swimming and rock climbing. Participant No. 13 said, “it generally decreases my ability to interact at home and enjoy life generally,” whereas Participant No. 9 explained how the injury impacted his social life, stating, “I was unable to connect with friends at the time”. Participant No. 1 explained the flow-on effects of injury:

*The biggest impact is not being able to participate in sports, and for that reason I have lost a lot of general fitness and tone and have put on about 15 kg over the past two years.*

Another important area influenced by injury was sleep. For example, “It affects my sleep; I wake up with mid-thoracic tightness at night” (Participant No 8). Others spoke of a complex set of impacts on their general health and energy levels. Participant No. 13 explained, “I get a bit of tension around the diaphragm, and it can be associated with a tension headache, lack of energy and lack of ability to concentrate.”

#### Theme 3: Seeking help

The third theme relates to participants’ experiences when seeking help from others and initiating self-help strategies to manage and rehabilitate from the injury. Help was sought in a variety of areas, including occupational therapy, pain specialist management including injections and medication for pain and inflammation, acupuncture, manual and exercise therapy (chiropractic, massage, osteopathy, physiotherapy), counselling and stress management, and attention to rest and nutrition. Two participants employed hand splinting in the form of strapping/bracing as an intervention to manage pain and as a preventative measure against further injury.

Some participants discussed a broader range of coping strategies that they used to navigate the psychological issues and stress associated with their injury. These included practising meditation or mindfulness; seeking regular de-briefing and support from counsellors and psychologists; establishing strong work–life boundaries; and having healthy habits and routines with regard to nutrition, exercise and sleep.

#### Theme 4: Injury prevention education in osteopathy

Most participants discussed the need for formal injury prevention training in pre-registration and continuing education courses for osteopaths. They suggested modules that focused specifically on the ergonomics of safe clinical practice, including biomechanics of force delivery and its effects through the practitioner’s body, effective and safe posture, and the use of whole body rather than strength. New graduates or those returning to practice after a long break need to build up a patient load slowly, be realistic about what can be achieved with each patient, and become informed of and take steps to reduce the risk of injury in clinical practice.

*Course content directed towards protecting osteopaths over decades of work…some specific physical health regimes about strengthening and stretching exercises set out for osteopaths that address the sort of injuries that we are likely to have. (Participant No. 9).*

Exercise physiology and exercise prescription courses were considered essential knowledge for graduating and practicing osteopaths. It was pointed out by several participants that in their opinion osteopathy is slow to engage with exercise as a primary treatment modality. They suggested in-depth knowledge of exercise physiology and experiential training in exercise therapy would equip osteopaths with the skills to design exercise rehabilitation programs for patients, reducing the hands-on manual therapy demands of osteopathic consultations. Such knowledge and skill would also benefit the practitioner to assess their own exercise and strengthening needs. It was acknowledged however that some osteopaths possess advanced exercise therapy skill, usually gained outside their osteopathic education.

*Traditionally our profession has not been good at applying non-manual therapy approaches, maybe [we are] less strong in our exercise advice and rehabilitation approaches. (Participant No. 4).*

*When osteopathy was developed, people moved [were active]. And that‘s why [Dr] Still treated trauma, not inertia. That‘s really no longer the case. It‘s taking the profession a really long time to come to grips with what the exercise literature is actually saying. It’s a difficult space because the consumers are really honing in on movement and as a profession, we are not really skilled up, at all, to really offer any meaningful clinical integration of movement and exercise into osteopathic practice (Participant No. 5).*

## Discussion

The current research explored the perceptions and experiences of 13 Australian osteopaths who had sustained a WRMI while undertaking clinical practice. The findings suggest that WRMIs can have numerous negative effects on the work life of osteopaths, necessitating their use of a variety of strategies to deal with their changed circumstances. The impact of injury also permeated life outside clinical practice, with pain and disability affecting sleep, driving, sporting and leisure activities and, in some cases, interactions with family and friends. In many instances, the osteopaths continued to work while injured. This finding is consistent with the findings from research involving PTs, OTs and chiropractors [5, 7, 9, 12, 18, 24]. For example, research by Darragh et al. [12] in a survey of 1158 PTs and OTs, found that almost half continued to work in clinical practice while injured. These authors suggest that working while injured or in pain—referred to as presenteeism—may stem from “the clinical culture of healthcare providers in which altruism is valued, so admitting an injury caused by patient care is difficult” (p. 359). They further posit that this presents not only the risk of further injury to the practitioner but also the risk of injury to the patient due to underperformance on the practitioner’s part. Others attribute the trend to work while injured to a perception on the part of therapists that sustaining an injury during patient care as a weakness, due to their expertise in managing patients with injury [9].

The most common cause of injury reported by the participants was repetitive high compressive and static load to joints and tissues, consistent with the findings from the quantitative arm of this research involving 160 osteopaths (McLeod G, Murphy M, Henare T, Dlabik B: Prevalence of work-related injuries in osteopaths: A preliminary investigation, submitted). Research involving other health professionals has also found sustained loading to body parts to be a risk factor for practitioner injury [5, 12, 15, 24, 25]. Participants offered numerous suggestions for injury prevention and risk minimisation that reflected their awareness of causative factors and the need for attention to safe work practices. These included an increased emphasis on risk minimisation in the curricula for students [26, 27] and experienced practitioners alike. All participants agreed that employing general self-care and health-seeking behaviours was key to minimising the risk of injury. Exercise, stretching, addressing poor ergonomic work postures and reducing prolonged and static loading were the most frequently mentioned strategies. However, from participants’ comments, it appears that risk minimisation strategies were often realised post injury. A similar finding was shown in research involving physical therapists, who also reported changing their work practices in response to injury but only in the presence of moderately severe symptoms [25].

WRMIs pose a number of concerns for the osteopathic profession. These concerns include the retention of workers in the profession [28]; patient safety; an absence of guidelines specific to osteopathic practice for workplace health and safety, including injury prevention and risk minimisation; legislative requirements pertaining to injury reporting; management of injury within the osteopathic workplace; and compensation issues. With their focus on the care of patients with musculoskeletal conditions, practitioners may not consider the risks of WRMI as applicable to themselves. Although most osteopaths would be aware of the risk of injury from physically demanding activities, WRMI among osteopaths has received little attention in the literature. However, Section 9 ‘Ensuring Practitioner Health’ in the Osteopathy Board of Australia Code of Conduct [29] does provide guidance for practitioner health. Explicit is the edict for the reporting of any condition that may interfere with patient care. Additionally, the Code advises practitioners to “…be aware of the risks of self-diagnosis and self-treatment” (p. 29) and of “recognising the impact of fatigue on practitioner health and ability to care for patients or clients and endeavouring to work safe hours whenever possible” (p. 29) [29].

Workplace health and safety is a core concern for Safe Work Australia, the national policy body responsible for improving occupational health and safety and workers’ compensation arrangements across Australia through the development of work health and safety laws [30]. The focus of these laws is high-risk workplaces, such as industries dealing with hazardous substances, electrical work or other dangerous environments where the risk of serious injury is present. However, there is a move to standardise the legislative requirement for all industries and professions to protect workers by ensuring safe work practices. Health care and social assistance is a priority for Safe Work Australia due to the high number of reported injuries [31]. A number of health professions—including nursing, medicine and physiotherapy—have undertaken preliminary work to develop guidelines and resources to reduce the risk of WRMI within the legislative framework [32–34]. As part of a comprehensive approach to understanding the incidence, prevalence, cause and management of WRMIs of Australian workers, the *Work Health and Safety Act 2011* includes the provision for notification and reporting of serious injury sustained at work. Strains and sprains however are not considered serious and do not warrant reporting under the Act [35].

Osteopathy is a small and an insular profession. The majority of osteopaths are self-employed and work in small practices that have limited resources to cover the workload of an injured practitioner. Although there is no empirical data on numbers of osteopaths who carry injury protection insurance, anecdotally few do. Apart from a very small number of work injury insurance claims (as described in this study), most WRMIs in osteopaths go unreported, and many practitioners find themselves alone in their struggle to continue working while managing their injury. The majority of osteopaths work in private practice, often with little or no collegial contact or support [36]. Such isolated work environments, coupled with a shortage of practitioners in many areas of Australia, places an added burden on practitioners who have sustained a WRMI—to continue to work while injured. Availability of locums for casual relief work in Australia is little to none. A recent search (August 2017) of the ‘Locum Wanted’ webpage hosted by Osteopathy Australia found not a single listing for an available locum, in contrast, there were 15 listings for positions vacant.

### Limitations and future research

As with all qualitative research, the findings cannot be generalised to a larger population. It cannot be assumed that the opinions expressed by the 13 osteopaths in the current research are representative of the experiences of all Australian osteopaths who have sustained and coped with a WRMI. Caution must be taken when interpreting the findings presented here because of the subjective nature of the individual participants’ accounts. Sampling bias posed a risk to the validity of the findings. The participants self-selected themselves on two occasions. Each of the participants initially responded to an online survey that gathered quantitative data, which was reported separately; at the conclusion of that survey, the participants consequently accepted an invitation to be involved in this research. Future research on the experiences of osteopaths with a WRMI might be better conducted using random selection.

The use of three researchers to conduct the interviews could pose the risk of researcher bias. Although a standardised interview question sheet was used by each researcher, the semi-structured nature of the interviews encouraged the interviewers— if they deemed it necessary to gain further information—to probe participants with additional questions. The decision to probe, or not, was at the discretion of the interviewer. All researchers received intensive interview training that included exposure to and practice with hypotheticals that would require the interviewer to initiate additional questioning. Opportunities to reflect critically on their unexamined expectations of, and personal motivation for, involvement in the research project highlighted for the researchers the risk of imposing their unconscious bias and agendas to the process of data collection, including question selection.

Further due diligence was undertaken by the researchers to limit researcher bias by using multiple analysts who applied repeated independent and consequent collaborative checking of the developing coding sheets. Additionally, reflexivity was central throughout the research process [37]. During team meetings and as part of the interviewer training sessions, the researchers reflected on and explored their own personal and shared biases towards qualitative and quantitative methodologies and their views about WRMIs. These methodological steps were considered essential to ensure trustworthiness in the findings.

Further research involving a greater representative sample is needed to validate and extend the findings. Wide-ranging stakeholder consultation to develop awareness of the risk of WRMIs including psychosocial work stressors in osteopathic practice is needed, together with the development of work safety guidelines for risk minimisation and injury prevention in osteopathy.

## Conclusion

The aim of this research was to explore the experiences and perceptions of a small number of Australian osteopaths who had sustained a WRMI and, consequently, to raise the awareness of the risk of WRMIs in osteopathic practice. We anticipate that this research will encourage a broad and constructive discussion within the profession of the issues associated with WRMIs, including risk minimisation and injury prevention, and will lead to further research and the development of guidelines and educational curricula addressing safe work for osteopaths.

## Appendix

### Exploring the experiences of Australian osteopaths who have sustained a workrelated musculoskeletal injury (WRMI)

#### Interview Questions

The interviewer will introduce themselves to the participant, provide a brief overview and purpose of the research and interview format and then ask permission to record the interview and gain written consent from the participant. The following questions will form the basis of the interview which will be undertaken in a semi-structured manner to allow the participant to provide as much relevant information as possible.

#### Demographics

- Where did you receive your osteopathic qualification?
- What year did you graduate?
- How many years have you been practicing osteopathy?
- How many patients do you see on average per day?
- How many days do you work a week?
- How long are your consultations with patients? (Initial/follow up)
- What amount of time do you allow between consultations?
- Practitioner health: rate your overall health from 1 to 10.
- How would you describe your treatments - direct, indirect etc.?
- Do you favour standing/seated techniques etc.?
- What adjunctive therapies do you personally use in practice? (dry needling, cupping, moxa, ultrasound, Clinical Pilates, yoga, aqua therapy, strapping)
- Do you have adjunctive therapists in your treatment regime? (describe)
- Are the adjunctive therapies combined in your allocated treatment time or are they contracting separately?

#### WRMSI

- In the survey you indicated that you have sustained a WRMI - can you please tell me a little bit about it?
- Please describe the location and the mechanism of your injury
- Was there a particular technique or posture that you believe caused or contributed to your injury? (repetitive techniques or single incident; HVLA, ergonomic/postural, sustained pressure)
- How many years had you been practicing when you acquired your injury/injuries?

#### Identifying WRMSI

- Was your WRMSI self-diagnosed or diagnosed by another health professional?
- What were the confirming factors or your diagnosis? (imagining, orthopaedic testing, google, bloods)
- What other factors may have contributed to your WRMSI?

#### Managing WRMSI

I like to know you managed your injury/injuries and the effect on your work and life

- Did you receive any treatment for this injury? What? For how long? Self-treatment?
- How often do you get musculoskeletal therapist treatments and what type?

#### Exploring the experiences of Australian osteopaths who have sustained a workrelated musculoskeletal injury (WRMI)

- What effect did your WRMSI have on your osteopathic practice? (time off work, how long, shorter work hours, leaving practice, ergonomic devices, work cover)
- What effect did your WRMSI have on your quality of life? (decreased or ceased leisure activities, effect on family life, financial stressors)
- What do you do differently now as a result of this injury?
- Does this WRMSI still effect your work?
- What activities do you do to manage your WRMSI?
- Are there any activities that you avoid?

#### Preventing WRMSI

- What injury prevention measures do you incorporate into your practice?
- What injury prevention measures do you incorporate into your life? (Exercise, strengthening, stretching, stress management)
- Is there prevention measures you would like to suggest related to your WRMSI?

#### Miscellaneous

- Do you feel your osteopathic educational institution placed awareness on injury prevention?
- What was/were your previous occupation/s and do you think they may have contributed any way to your current injury/injuries?
- Do you have any words of wisdom to share with the osteopathic community in regard to WRMSI?
- Is there anything else you feel is relevant?

## Abbreviations

OT: Occupational therapist
PT: Physical therapist/Physiotherapist
WRMI: Work-related musculoskeletal injury
